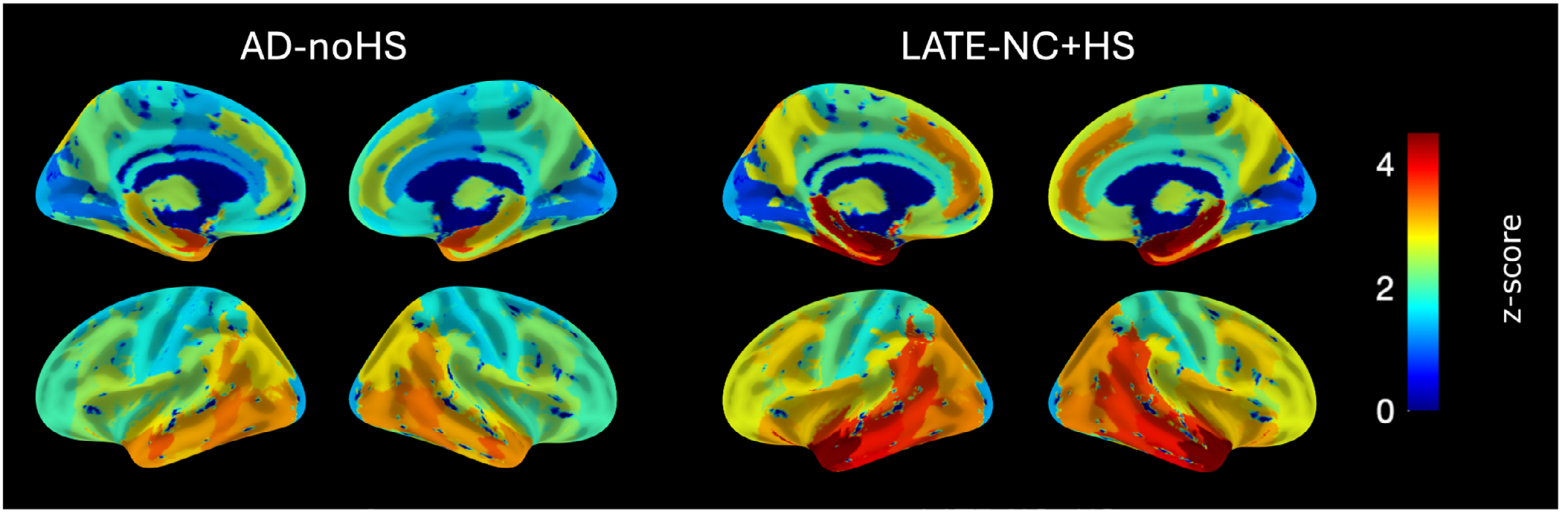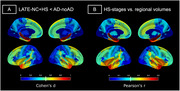# The MRI signature of LATE‐NC‐associated hippocampal sclerosis extends beyond the medial temporal lobe

**DOI:** 10.1002/alz70862_110866

**Published:** 2025-12-23

**Authors:** Michel J. Grothe, Linda Zhang, Francisco J. López‐González, Pascual Sanchez‐Juan, Alberto Rabano, Jesús Silva‐Rodríguez

**Affiliations:** ^1^ CIEN Foundation, Reina Sofia Alzheimer Center, ISCIII, Madrid, Madrid Spain

## Abstract

**Background:**

Limbic age‐related TDP‐43 encephalopathy neuropathologic change (LATE‐NC) is often comorbid to Alzheimer’s disease neuropathologic change (ADNC) and it is linked to hippocampal sclerosis (HS), which contributes to amnestic deficits independently of AD. Previous imaging‐pathologic association studies have described a distinct temporo‐limbic FDG‐PET signature of HS that helps to differentiate it from AD. MRI‐based studies have described severe hippocampal atrophy in HS, but the brain‐wide atrophy pattern of LATE‐NC‐associated HS remains to be characterized in more detail.

**Methods:**

We studied 84 amnestic dementia patients from our brain bank cohort, who underwent detailed neuropathologic assessment at autopsy and had quality‐controlled ante‐mortem 3T‐MRI available (MRI‐to‐death interval: 3.0±3.0 years). Neuropathologic evaluation included standard assessments of ADNC and LATE‐NC, and HS was assessed using a standard binary approach (present/absent) as well as a novel pathologic staging scheme considering four consecutive stages of HS severity. MRI data was processed using CAT12/SPM12 and analyzed using ROI‐based analyses across 52 cortical and subcortical brain regions. Regional volumes of autopsy cases were compared to normative data of a sample of 1032 healthy elderly controls scanned on the same scanner.

**Results:**

Compared to controls, dementia cases with LATE‐NC+HS (*N* = 30) and those with typical AD (ADNC>=2) without HS (AD‐noHS; *N* = 47) had highly similar brain‐wide patterns of atrophy, being most pronounced in the medial and anterior temporal lobe, followed by lateral temporo‐parietal areas (Fig‐1). In a direct comparison between pathologic groups, LATE‐NC+HS cases had significantly more pronounced atrophy in the hippocampus and amygdala, but also in more extended areas of the temporal and frontal lobes, including the temporal pole, insula, and orbitofrontal cortex (Fig‐2A). A largely identical HS‐associated temporo‐limbic atrophy pattern was observed when correlating the pathologic staging scheme of HS severity with regional brain volumes across all autopsy cases (Fig‐2B). No significant differences were observed between AD‐noHS cases with (*N* = 24) vs without comorbid LATE‐NC (*N* = 23).

**Conclusions:**

LATE‐NC‐associated hippocampal sclerosis is linked to an in‐vivo atrophy pattern on MRI that extends well beyond the hippocampus and includes several temporo‐limbic cortical areas known to accumulate TDP‐43 pathology in LATE‐NC. This signature could help to improve clinical detection of this pathologic condition in‐vivo.